# LiqMat: Liquidity-Enabled Matters for Adaptive Electronics

**DOI:** 10.34133/research.1442

**Published:** 2026-09-22

**Authors:** Sen Chen, Jing Liu

**Affiliations:** ^1^ State Key Laboratory of Mechanics and Control for Mechanical Structures, Institute for Frontier Science, Nanjing University of Aeronautics and Astronautics, Nanjing, China.; ^2^ Key Laboratory of Cryogenic Science and Technology, Technical Institute of Physics and Chemistry, Chinese Academy of Sciences, Beijing, China.

## Abstract

Life chooses liquids, whereas modern electronics has long relied on solids. This fundamental distinction has shaped 2 remarkably successful yet largely independent information systems: biological systems that achieve adaptation through dynamic liquid environments and electronic systems that rely on static solid-state architectures. Although flexible electronics have largely improved mechanical compliance, their functionality remains predominantly encoded in fixed conductive pathways and predefined interfaces, limiting their ability to adapt. Here, we introduce LiqMat (liquidity-enabled matters) as a materials paradigm for adaptive electronics. Rather than defining a specific material class, LiqMat describes functional material systems whose properties emerge from liquidity-enabled dynamics rather than relying solely on static structures. We propose that dynamic interfaces, adaptive transport, and structural evolution constitute 3 core characteristics of LiqMat. These characteristics are shared by diverse liquid-enabled material systems, including aqueous systems and hydrogels, ionic liquids and ionogels, and electrofluids, as well as liquid metals and their composites, despite their distinct chemistries and transport mechanisms. Of these, liquid metals provide a particularly illustrative example of LiqMat by combining high electronic conductivity with fluidic adaptability. Looking forward, LiqMat provides a conceptual framework for shifting electronics from mechanically flexible yet functionally static systems toward adaptive electronics inspired by the dynamic organization of living systems.

## Introduction

Life and electronics represent the 2 most successful information systems on earth, yet they are built upon fundamentally different material foundations. Biological systems emerge, evolve, and function in liquids. Water not only serves as the medium for life but also actively participates in ion transport, molecular communication, metabolic regulation, and self-organization [1]. In contrast, modern electronics has developed almost entirely upon solid-state materials. From silicon semiconductors to integrated circuits, electronic functionality has traditionally relied on stable crystal structures, fixed interfaces, and deterministic charge transport. These 2 strategies have independently driven extraordinary advances in biology and information technology.

As electronics increasingly moves beyond traditional computing toward wearable devices, bioelectronics, soft robotics, and human–machine interfaces, this solid-state paradigm begins to encounter its limitations [2]. Flexible electronics has substantially improved the mechanical compatibility between electronic devices and soft biological tissues through stretchable conductors, deformable substrates, and structural engineering [2,3]. However, despite their mechanical flexibility, most existing flexible electronic systems remain fundamentally static in function. Their conductive pathways, interfaces, and architectures are largely predetermined during fabrication and are expected to remain unchanged throughout operation. Consequently, many defining characteristics of living systems, such as autonomous adaptation, dynamic reconfiguration, transformable tuning, and self-recovery, remain difficult to achieve via conventional material concepts alone. These observations suggest that the central challenge may not simply be making solid materials softer, but rather exploiting the unique capabilities enabled by liquids. Unlike solids, liquids continuously reorganize their interfaces, redistribute matter and charge, and evolve toward energetically favorable configurations. Such dynamic behaviors provide an alternative route for generating functionality that cannot be readily realized in traditional static materials.

**Fig. 1.**
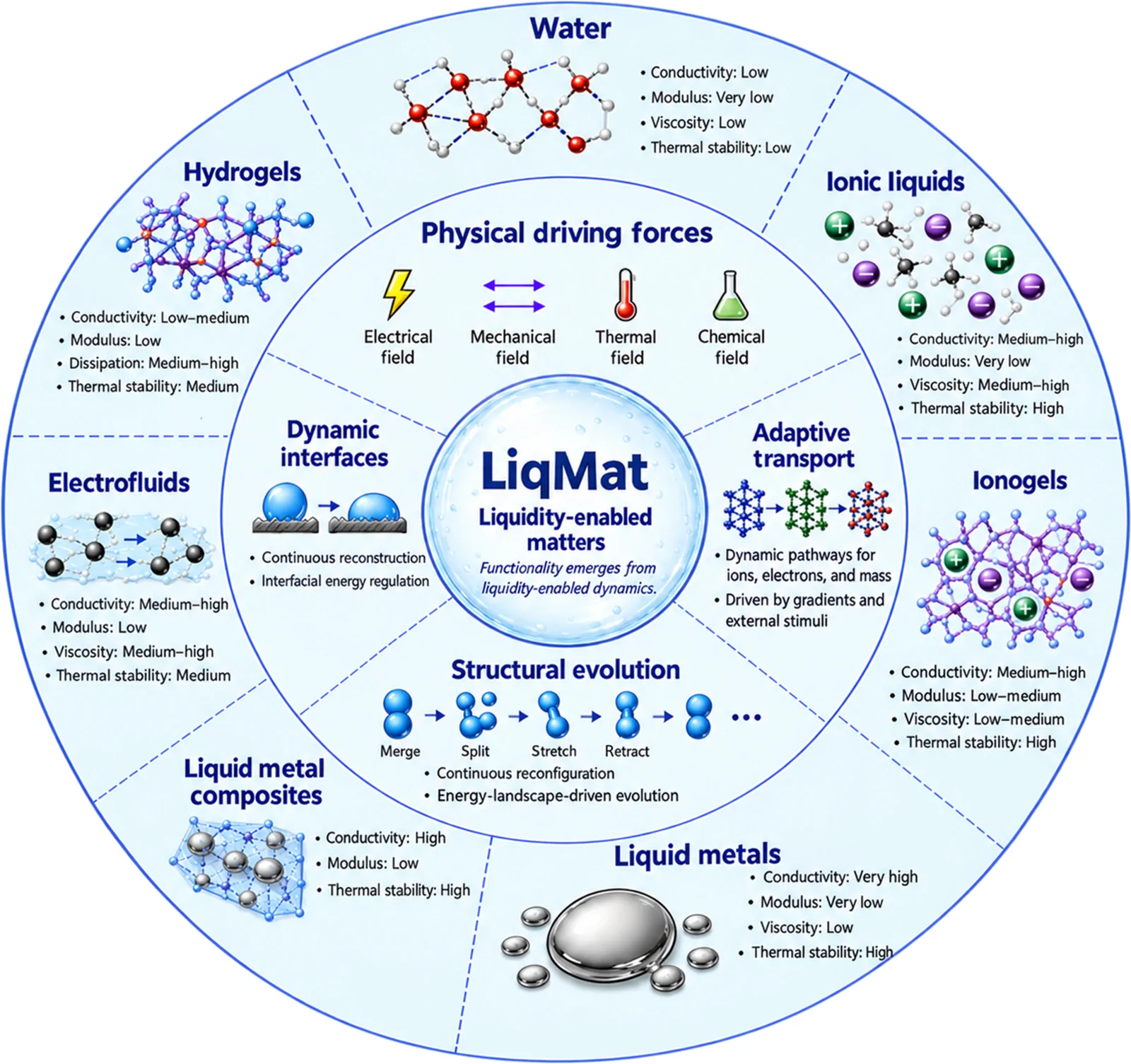
Conceptual framework of liquidity-enabled matters (LiqMat). The framework illustrates how external physical driving forces activate liquidity-enabled dynamics, giving rise to 3 core characteristics: dynamic interfaces, adaptive transport, and structural evolution. These characteristics are exemplified across representative LiqMat platforms, including water, hydrogels, ionic liquids, ionogels, liquid metals and their composites, and electrofluids, and are linked to their characteristic material properties and resulting electronic functionalities.

Here, we introduce LiqMat (liquidity-enabled matters) as a materials-oriented conceptual framework in which liquidity is treated as a functional design principle for generating adaptive electronic behavior (Fig. 1). Unlike existing reviews that survey hydrogels, ionogels, or liquid metals separately, this perspective focuses on the common functional logic that cuts across these platforms, namely, liquidity-enabled adaptation. LiqMat complements established soft-matter concepts by focusing specifically on how liquid-enabled dynamics can be exploited as a source of electronic functionality. By harnessing these dynamics, LiqMat provides a distinct pathway for functional emergence, establishing a conceptual foundation for the next generation of adaptive electronic materials.

## What Is LiqMat?

Classical electronic materials derive functionality from fixed chemical compositions and static structures. Once fabricated, stable electrical behavior is anticipatable, with performance optimization focused on structural precision and the suppression of operational instabilities. This philosophy underpins the success of solid-state electronics. LiqMat represents a fundamentally different materials paradigm. Rather than encoding functionality solely within static structures, LiqMat derives functionality from the dynamic behaviors enabled by liquidity. Accordingly, we define LiqMat as a materials framework in which liquidity-enabled dynamics give rise to adaptive functionality through intrinsic material reorganization that helps maintain, restore, or improve function as operating conditions change. Within this framework, liquidity is regarded not merely as a physical state but as a functional design principle capable of generating adaptive material behaviors.

The core characteristics of LiqMat arise from the intrinsic dynamics of liquid or liquid-like systems. Physical stimuli such as mechanical deformation, electric fields, thermal gradients, and chemical environments, coupled with the intrinsic mobility of liquids, drive the redistribution of matter and charge that gives rise to dynamic interfaces, adaptive transport, and structural evolution in LiqMat systems. Unlike rigid solid interfaces, liquid-enabled interfaces continuously reconstruct through wetting, molecular rearrangement, adsorption, and interfacial transport, processes governed by local interfacial energy regulation rather than global energy minimization. Consequently, the interface itself becomes an active functional component that dynamically regulates electrical coupling, mechanical contact, and interactions with surrounding environments. At the same time, transport within LiqMat is inherently adaptive rather than fixed. Electrons, ions, molecules, and even the liquid phase itself can continuously redistribute under mechanical, electrical, thermal, or chemical stimuli, allowing transport pathways to reorganize during operation in response to changing environmental conditions. The dynamic nature of LiqMat further extends to its structural organization. Whereas conventional electronic materials generally preserve fixed structural configurations after fabrication, LiqMat systems continuously reorganize through fluid flow, interfacial interactions, phase transitions, or network reconstruction, reflecting energy-landscape-driven evolution rather than simple relaxation toward equilibrium. Material structure therefore becomes an evolving variable instead of a predetermined framework, enabling reconfigurable conductive networks and adaptive material functions.

Together, dynamic interfaces, adaptive transport, and structural evolution constitute the 3 core behavioral characteristics of LiqMat (Fig. 1). These characteristics should be understood collectively rather than as independent or exclusive classification criteria, as each may also arise in nonliquid systems, but rarely with the same degree of continuous, fluidic reconfigurability. Within LiqMat, their importance lies in their common origin in liquidity-enabled mobility and continuous material reorganization, which are functionally coupled to electronic, ionic, or mass transport and support adaptive functionality. While established concepts such as soft, reconfigurable, stimuli-responsive, adaptive, active, and dynamic materials each emphasize distinct attributes, including mechanical compliance, structural reorganization, environmental responsiveness, or energy-driven activity, LiqMat focuses on liquidity as a functional design principle that connects these dynamic processes across diverse material platforms. In this way, functionality is not solely encoded in stable structures but can emerge and evolve through liquidity-enabled dynamics.

## Representative LiqMat Platforms

The concept of LiqMat is not restricted to a specific chemical composition or a single type of liquid phase. Instead, it provides a broader conceptual framework encompassing material systems in which functionality emerges from liquidity-enabled dynamics. Different material platforms achieve such dynamic behaviors through distinct mechanisms. Here, we highlight several representative LiqMat platforms that exemplify how liquidity can serve as a fundamental source of functionality (Fig. 1).

### Water-based systems and hydrogels

Water-based materials represent one of the most fundamental manifestations of LiqMat because water provides a dynamic environment for molecular transport, ion conduction, and biological interactions [1,4]. Hydrogels, consisting of hydrated polymer networks, combine structural integrity with liquid-like molecular mobility, enabling continuous exchange of ions, molecules, and chemical signals [4,5]. Their dynamic hydration states allow interfaces to adapt to external stimuli, while ionic transport through aqueous pathways enables communication across soft material systems. These characteristics make water-based LiqMat platforms particularly relevant for biointerfaces and biomimetic electronics [6,7], where information processing relies on dynamic interactions rather than rigid architectures. They demonstrate how liquidity can support adaptive functions analogous to those found in biological systems.

### Ionic liquids and ionogels

Ionic liquids and ionogels provide another important LiqMat platform by utilizing mobile ionic species as functional carriers. Unlike established solid electrolytes, these materials maintain highly dynamic ionic environments while preserving mechanical stability through polymer or network confinement [8]. The redistribution of ions under external stimuli enables adaptive electrical double-layer formation, interfacial modulation, and electrochemical coupling. Such dynamic ionic behaviors have established ionogels as promising materials for soft iontronic systems [9]. More importantly, they reveal that interfaces are not merely passive boundaries but active regions where functionality continuously emerges through ionic rearrangement.

### Liquid-metal-based adaptive systems

Among various LiqMat platforms, liquid metals represent one of the most representative platforms of the concept by uniquely integrating metallic conductivity with liquidity-enabled adaptability [10,11]. Unlike classical metals with fixed structures, liquid metals can continuously reconfigure their morphology while preserving efficient electrical transport [12]. Their adaptive behavior arises not only from bulk fluidity, which enables shape transformation and reconfigurable conductive pathways [13], but also from dynamically evolving oxide-mediated interfaces that regulate wetting, adhesion, interfacial charge transfer, and structural stability. Together, these features endow liquid metals with remarkable responsiveness to external stimuli [14], including electric, magnetic, acoustic, optical, and thermal fields, allowing their structures, interfaces, and transport properties to be programmably modulated. In composite systems, these capabilities can be further expanded through interactions with polymers [15,16], particles [17], and other functional phases, creating hybrid materials in which electrical pathways, mechanical properties, and system-level functionalities coevolve. Liquid metals therefore exemplify how liquidity can serve not merely as a physical state, but as an active design parameter for adaptive electronic functionality.

### Electrofluids and beyond

Electrofluids represent an emerging LiqMat platform in which conductive particles are dispersed within insulating liquid matrices [18,19]. Their electrical properties arise from dynamically evolving percolation networks, where particle contacts continuously form and break during deformation or flow, producing coupled rheological and electrical responses. This dynamic coupling directly embodies the structural evolution and adaptive transport that define LiqMat, placing electrofluids between conventional solid conductive composites and liquid metals and offering a distinct route toward flowable, stretchable, and reconfigurable electronics [20]. Electrofluids further illustrate the expanding scope of LiqMat, which is not limited to the representative platforms discussed here but can encompass emerging liquid-based conductive systems that exhibit these core characteristics.

These platforms represent complementary realizations of LiqMat with distinct functional trade-offs. Hydrogels and ionogels favor ionic transport and biointegration, electrofluids enable dynamically reconstructed conductive networks, while liquid metals offer fluidity with high electronic conductivity. Thus, LiqMat platform selection should be function driven, requiring a balance among conductivity, stability, processability, and biointegration. Rather than functioning as isolated material platforms, these platforms can also be integrated into hybrid ionic–electronic systems. Within these systems, ions provide bioinspired mechanisms for adaptive communication, environmental responsiveness, and dynamic regulation, whereas electrons deliver the high-speed, high-efficiency transport required for modern electronics. In this sense, ions enable electronics to learn from life, while electrons allow adaptive systems to transcend the performance constraints of purely biological information processing. Their integration represents one of the central ideas of LiqMat, establishing a unified materials platform that bridges biological principles with next-generation adaptive electronics.

## From LiqMat to Adaptive Electronics

As electronic systems progressively move into complex environments such as biological tissues, soft robots, and human–machine interfaces, future devices must operate under conditions that are far more dynamic and unpredictable than those encountered in traditional electronics. In such scenarios, mechanical flexibility alone is insufficient to ensure reliable functionality. Instead, future electronics must inherently acquire the ability to dynamically regulate function, reorganize internal states, and maintain performance under continuously changing conditions. These demands point toward a broader transition from flexible electronics to adaptive electronics.

From the perspective of LiqMat, this transition can be understood through 4 fundamental shifts. First, electronics must evolve from static to dynamic, where functional states are no longer permanently defined during fabrication but can continuously evolve during operation. Second, systems must transition from fixed to reconfigurable, enabling conductive pathways, interfaces, and transport networks to reorganize in response to internal or external perturbations. Third, device behavior must advance from passive to adaptive, such that materials actively participate in functional regulation rather than merely responding to external control signals. Finally, future electronic systems may progress from rigid to living-like, progressively exhibiting properties such as self-regulation, self-recovery, and long-term environmental adaptation that are characteristic of biological systems.

However, the liquidity that enables reconfiguration and adaptation also introduces trade-offs in stability, controllability, and processability. Thus, LiqMat should aim not to maximize liquidity, but to balance these attributes according to the target function. Key challenges include the quantitative description and prediction of adaptive transport in evolving percolation networks, the programming of dynamic interfaces, and the interplay among thermodynamics, kinetics, dissipation, and external driving forces. Equally important are long-term encapsulation of liquid phases, integration with conventional manufacturing, and energy efficiency in actively driven systems. A further conceptual challenge is to determine when a dynamic response becomes genuine functional adaptation and how adaptation can be quantitatively distinguished from simple responsiveness or externally programmed reconfiguration. Addressing these questions will be essential for translating LiqMat into scalable technologies and for developing predictive design principles for adaptive electronics. Taken together, these challenges highlight the broader opportunity offered by LiqMat. By harnessing liquidity-enabled reorganization, LiqMat provides a materials-oriented route toward electronics in which functionality can evolve rather than remain entirely predefined.

## Conclusion

Electronics in the past century has been fundamentally constructed upon solid-state materials, where functionality is encoded through fixed atomic arrangements and predefined device architectures. This paradigm has enabled remarkable technological progress, but it also imposes intrinsic limitations on adaptability and long-term functional evolution. The LiqMat concept suggests an alternative design principle in which liquidity is not treated as a constraint to be eliminated, but as an active resource for generating functionality. By enabling dynamic interfaces, evolving transport pathways, and continuously reconfiguring structures, liquidity introduces a distinct mechanism for functional emergence that complements traditional solid-state approaches. Rather than replacing existing electronic technologies, LiqMat expands the conceptual and materials space for electronics that are not merely flexible, but dynamically reconfigurable.

More broadly, it provides a bridge between biological and electronic systems, where functionality is not solely defined by structure, but continuously shaped by dynamics. Looking ahead, the integration of ionic and electronic pathways within a single liquid-enabled platform may enable new forms of bioinspired computation and sensing that are difficult to achieve with rigid architectures. The ability to program interfacial dynamics through external fields could further lead to materials whose functions can be tuned in real time, while hybrid systems combining liquid metals, hydrogels, ionogels, and electrofluids may eventually bridge the gap between high-performance electronics and biological compatibility. Realizing these possibilities will require continued progress in stability, encapsulation, and scalable manufacturing, but these challenges define important directions for establishing LiqMat as a predictive framework for adaptive electronics.
